# Prognostic factors of atrial fibrillation following elective coronary artery bypass grafting: the impact of quantified intraoperative myocardial ischemia

**DOI:** 10.1186/1749-8090-6-127

**Published:** 2011-10-03

**Authors:** Efstratios N Koletsis, Christos Prokakis, James R Crockett, Panagiotis Dedeilias, Matthew Panagiotou, Nikolaos Panagopoulos, Nikolaos Anastasiou, Dimitrios Dougenis, Efstratios Apostolakis

**Affiliations:** 1 Cardiothoracic Surgery Department, University of Patras, School of Medicine, Patras, Greece; 2 1st Cardiac Surgery Department. "Evangelismos" General Hospital, Athens, Greece; 3 Cardiac Surgery Department, Athens Medical Center, Greece; 4 Department of Thoracic Surgery, 1st IKA Hospital, Athens, Greece; 5 Department of Cardiac Surgery, University of Ioannina, School of Medicine, Ioannina, Greece

**Keywords:** post-CABG atrial fibrillation, cardiopulmonary bypass, coronary artery bypass grafting, CABG, Myocardial Ischemia Index, postoperative supraventricular arrhythmias, predictors

## Abstract

**Background:**

Atrial fibrillation (AF) occurs in 28-33% of the patients undergoing coronary artery revascularization (CABG). This study focuses on both pre- and peri-operative factors that may affect the occurrence of AF. The aim is to identify those patients at higher risk to develop AF after CABG.

**Patients and methods:**

Two patient cohorts undergoing CABG were retrospectively studied. The first group (group A) consisted of 157 patients presenting AF after elective CABG. The second group (group B) consisted of 191 patients without AF postoperatively.

**Results:**

Preoperative factors presenting significant correlation with the incidence of post-operative AF included: 1) age > 65 years (p = 0.029), 2) history of AF (p = 0.022), 3) chronic obstructive pulmonary disease (p = 0.008), 4) left ventricular dysfunction with ejection fraction < 40% (p = 0.015) and 5) proximal lesion of the right coronary artery (p = 0.023). The intraoperative factors that appeared to have significant correlation with the occurrence of postoperative AF were: 1) CPB-time > 120 minutes (p = 0.011), 2) myocardial ischemia index < 0.27 ml.m^2^/Kg.min (p = 0.011), 3) total positive fluid-balance during ICU-stay (p < 0.001), 4) FiO_2_/PO_2 _> 0, 4 after extubation and during the ICU-stay (p = 0.021), 5) inotropic support with doses 15-30 μg/Kg/min (p = 0.016), 6) long ICU-stay recovery for any reason (p < 0.001) and perioperative myocardial infarction (p < 0.001).

**Conclusions:**

Our results suggest that the incidence of post-CABG atrial fibrillation can be predicted by specific preoperative and intraoperative measures. The intraoperative myocardial ischemia can be sufficiently quantified by the myocardial ischemia index. For those patients at risk we would suggest an early postoperative precautionary anti-arrhythmic treatment.

## Background

Atrial Fibrillation (AF) remains the most common arrhythmia after cardiac surgery. Its incidence depends on patient's preoperative profile and the type of operation performed. AF occurs in approximately 28-33% of the patients undergoing coronary artery bypass grafting (CABG) [1-3] and in 30-63% of those operated for coexisting ischemic heart and valve disease [3,4]. The majority of AF arrhythmias appear within the first 4-5 postoperative days and the peak frequency is in the 2^nd ^or 3^rd ^postoperative day [5,6]. It has been reported that patients with postoperative AF have longer Intensive Care Unit (ICU) stay, longer hospitalization, and higher incidence of re-admissions increasing the cost of hospitalization by 30% [3,7]. This study is focused on the definition or pre- and peri-operative factors associated with the development of AF after CABG. The primary point is to find those patients at increased risk that may benefit of a precautionary preoperative anti-arrhythmic treatment.

## Materials and methods

### Patients

From 2002 to 2006 514 patients were operated on for coronary artery disease at the 1^st ^Cardiac Surgery Department at "Evangelismos" General Hospital in Athens, and the Cardiothoracic Surgery Department at Patras University. One hundred and sixty six patients were excluded from further analysis because of the following exclusion criteria: 1) preoperative, chronic (duration > 3 months) AF, 2) anti-arrhythmic treatment or history of cardiac arrhythmia other than AF, 3) concomitant heart valve disease other than trivial to mild ischemic mitral regurgitation (1+ or 2+/4+), 4) significant ischemic mitral regurgitation requiring mitral valve repair or substitution, 5) abnormal thyroid function or treatment for any thyroid disease, 6) acute or chronic renal failure (creatinine levels ≥ 200 mMol/L), 7) symptomatic congestive heart failure or severe dysfunction of the left ventricle (EF ≤ 0.30), 8) administration of any other medication except those for coronary disease (b-blockers, nitrates, calcium channel blockers, and anti-platelets), 9) history of previous neurologic stroke or deficit, and 10) re-operation. The remaining 348 patients were divided in two groups. The first group (Group A) included 157 patients (45.12%) undergoing CABG who developed postoperative AF within the first 10 postoperative days. The treatment of AF consisted of medical therapy and/or electrical cardioversion. The second group (Group B) included 191 patients (54.88%) having the same operation but without the occurrence of postoperative AF. Methods and treatments were the same in both study groups. Oral anti-anginal medication was continued until the day of operation unless unstable angina was present. In this case continuous intravenous anti-anginal treatment was given until surgery.

### Surgical procedure

All patients were operated on cardiopulmonary bypass. The distal anastomoses were performed first. The left internal mammary artery (LIMA) was exclusively used to bypass left anterior descending artery (LAD) stenoses whenever it was chosen as suitable (flow > 60 ml/min and sufficient length). Major saphenous vein grafts were used to bypass the diseased marginal (OM), diagonal (Diag) and/or right coronary artery (RCA). The proximal anastomoses were constructed during re-warming with the aorta de-clamped. Just after discontinuation of cardiopulmonary bypass and thereafter, in the ICU, a fluid-balance was daily recorded. During the ICU-stay and later on, in the ward, the ratio FiO_2_/pO_2 _was recorded to estimate the grade of hypoxemia. All patients were under surveillance in the ICU for the first 24-72 hours. Further observation for any arrhythmia development was carried out in the ward till discharge. When episodes of AF appeared, treatment consisted in amiodarone infusion with or without electrical cardioversion.

### Myocardial protection

Myocardial protection was obtained using systemic hypothermia (28°-30°C) and intermittent administration of cold blood cardioplegia. Initial infusion of cardioplegia was 1000 ml through the aortic root (antegrade). Thereafter it was infused via the coronary ostia and/or the grafts (after the completion of each distal anastomosis), in repeated doses of 300-400 ml at target intervals of 15-20 minutes. The pressure of cardioplegic perfusion was 100 mmHg, the temperature of cardioplegic solution was 6-8° Celcius, and the infusion flow was 250 ml/min. Therefore, the total volume of cardioplegia was mainly depended on the number of the distal anastomoses performed and generally on the length of aortic cross-clamp time. We estimated the myocardial injury related to myocardial protection by applying a mathematic model which included some factors known to present a strong relation with the development of AF: volume of cardioplegia, time between each cardioplegic delivery, temperature and body mass index. We called the final measure of this model the Myocardial Ischemia Index (MII) and it was estimated as follows:

MII ∞ [V_C _× F_C _× (P_D_-P_S_)]/[B.M.I. × I.i. × T_C_], where:

1) V_C _= volume of cardioplegia)

2) F_C _= cardioplegia flow; fixed at 250 mls/min. by protocol

3) T_C _= cardioplegia temperature; fixed at 6°C by protocol

4) (P_D_-P_S_) = cardioplegia delivery pressure minus coronary sinus pressure; fixed by protocol at 100 mmHg

5) I.i. = ischemia interval; time between each cardioplegia delivery for each anastomosis performed

6) B.M.I. = Body Mass Index; relative approximation to cardiac muscle mass.

Thus, considering that F_C_, (P_D_-P_S_) and T_C _were constant and fixed by the protocol, this leaves us with the approximation:

MII ∞ V_C_/(B.M.I. × I.i.) with the units expressed in mls.m^2^/kg.min.

The MII was calculated, using this more abbreviated approximation, for each antegrade delivery and it was termed MII_ante_. For each patient both the minimum value (_min_MII_ante_) and the average one (_av_MII_ante_) resulting from the sum of the values for patient were calculated.

### Postoperative indices of myocardial infarction

The levels of serum myocardial enzymes (CK, CK-MB) were daily checked after surgery. Troponin I levels were not routinely checked. The diagnosis of myocardial infarction (MI) was based on the ECG alterations, the level of the enzymes and the results of cardiac echo. ORS widening persisting for more than 12 hours after surgery or new Q wave combined with positive enzyme values and echo evidence of new focal disturbances in myocardial performance pointed out the occurrence of perioperative MI.

### Statistical analysis

All values are expressed as mean ± standard deviation. Comparison of data among the two groups of patients was performed by the Pearson chi square test (asym 2-sided) and the Fischer exact test. Values less than 0.05 were considered statistically significant. All analyses were performed using the SPSS 16 statistical package.

## Results

Tables 1 and 2 describe the patients' preoperative and main intra and post-operative characteristics respectively.

**Table 1:** Patients' clinical and preclinical characteristics

Clinical characteristics	Number of patients	Percentage
**Gender**		
Male	297	85.30%
Female	51	14.70%

**Age: 62.2 ± 9 (43-82 years)**		
41-55	83	23.90%
56-65	136	39.05%
> 65	129	37.05%

**Diabetes**	49	14.10%

**History AF (<3 months)**	48	13.80%

**History MI**	131	37.70%
Anterior MI	89	25.60%
Posterior MI	42	12.10%
		

**COPD**	44	12.60%

**OPA**	48	13.80%

**Unstable angina**	30	8.60%

**Obesity (BMI > 30)**	43	12.40%

**Hypertension**	151	43.40%

**Preclinical characteristics**		

**Diseased vessels**		
CAD-1	19	5.50%
CAD-2	56	16.10%
CAD-3	270	77.60%
LMCAD	34	9.80%

**E.F**		
0.30-0.40	53	15.20%
0.40-0.55	64	18.40%
> 0.55	231	66.40%

**Mild MR**	22	6.30%

**L.A dilation (> 40 mm)**	26	7.50%

**Proximal stenosis**		
Proximal LAD	81	23.30%
Proximal LCx	114	32.80%
Proximal RCA	74	21.30%

**Dyslipidemia**	189	54.30%

**Medical treatment**		
Nitrates	296	85.10%
b-blockers	258	74.10%
Ca^++^blockers	143	41.10%
Anti-platelets	284	81.60%

AF: atrial fibrillation, MI: myocardial infarction, COPD: chronic obstructive pulmonary disease, OPA: obstructive peripheral arteriopathy, BMI: body mass index, CAD: coronary artery disease, E.F: ejection fraction, MR: mitral regurgitation, L.A: left atrium, LAD: left anterior descending artery, LCx: circumflex artery, RCA: right coronary artery

**Table 2:** Patients' intra and postoperative characteristics

Characteristic	Number of patients	Percentage
**CPB-time: 98 ± 13 min (43-158)**		
CPB-time < 60	35	10%
CPB-time 60-120 min	237	68.10%
CPB-time > 120 min	76	21.90%

**Ischemia time: 47 ± 16 min (16-79)**		

**Myocardial Ischemia Index (M.I.I): 0.1- 1.0 ml.m^2^/Kg.min**		
av.MII _ante _≥ 0.5 ml.m^2^/Kg.min	104	29.90%
av.MII _ante _0.28 - < 0.49 ml.m^2^/Kg.min	176	50.60%
av.MII _ante _≤ 0.27 ml.m^2^/Kg.min	68	19.50%

**Bypasses performed**		
CABG-1	24	6.90%
CABG-2	102	29.30%
CABG-3	210	60.40%
CABG-4	12	3.40%

**LIMA use**	312	89.70%

**Positive fluid balance**	207	59.50%

**Potassium Deficit**	276	79.30%

**FiO_2_/PO_2_**		
≤ 40	302	86.80%
> 40	46	13.20%

**Inotropic support**		
Absent	235	67.50%
3-15 μg/kg/min	62	17.80%
> 15 μg/kg/min	51	14.70%

**Perioperative myocardial infarction**	19	5.50%

ICU-recovery		
≤ 48 hours	279	80.20%
> 48 hours	69	19.80%

CPB: Cardiopulmonary Bypass, M.I.I: Myocardial Ischemia Index, CABG: Coronary Artery Bypass Grafting, LIMA: left internal thoracic (mammary) artery, FiO_2_: fraction of delivered O_2_, PO_2_: arterial partial pressure of O_2_, ICU: Intensive Care Unit

The incidence of postoperative atrial fibrillation for the total cohort of patients was 45.1% (157 out of 348 patients). Comparing the two groups of patients in relation to their preoperative characteristics we found that the parameters having statistically significant impact on the postoperative occurrence of AF were the following (table 3): 1) age > 65 (p = 0.029), 2) history of AF (p = 0.022), 3) chronic obstructive pulmonary disease (p = 0.008), 4) left ventricular dysfunction expressed by EF < 0.40 (p = 0.015) and 5) proximal RCA stenosis (p = 0.023). The intra-, and post-operative parameters statistically related to the occurrence of postoperative AF were (table 4): 1) CPB-time above 120 minutes (p = 0.011) (cross clamp time not statistically significant, p < 0.05) 2) _av_MII_ante _value less than 0.27 ml.m^2^/Kg.min (p = 0.011), 3) positive fluid balance during ICU recovery (p < 0.001), 4) FiO_2_/pO_2 _ration ≤ 0.40 during ICU stay (p = 0.021), 5) high dose (> 15 μg/Kg/min) inotropic support (p = 0.016), and ICU-stay > 48 hour for any reason (p < 0.001).

**Table 3:** Impact of patients' preoperative characteristics on the development of post-CABG atrial fibrillation

Characteristic	Group A (AF)	Group B (no AF)	Significance (p)
	157 patients	191 patients	
**Gender (male vs female)**			p = 0.359
Male	137	160	
Female	20	31	

**Age**			
41-55	33	50	NS
56-65	56	80	NS
> 65	68	61	p = 0.029

**Diabetes**	25	24	NS

**History AF**	29	19	p = 0.022

**History MI**			
Anterior MI	41	48	NS
Posterior MI	18	24	

**COPD**	28	16	p = 0.008

**OPA**	19	29	NS

**Unstable angina**	12	18	NS

**Obesity (BMI > 30)**	24	19	NS

**Hypertension**	72	79	NS

**Diseased vessels**			
CAD-1	8	11	NS
CAD-2	26	30	
CAD-3	123	147	
LMCAD	16	18	

**E.F**			
0.30-0.40	32	21	p = 0.015
0.40-0.55	25	39	NS
> 0.55	100	131	NS

**Mild MR**	12	10	NS

**L.A dilation (> 40 mm)**	11	15	

**Proximal stenosis**			
Proximal LAD	43	38	NS
Proximal LCx	55	59	NS
Proximal RCA	42	32	p = 0.023

**Dyslipidemia**	85	104	NS

**Medical therapy**			
Nitrates	137	159	NS
Β-Blockers	115	143	
Ca^++^- blockers	68	75	
Anti-platelets	131	153	

AF: atrial fibrillation, MI: myocardial infarction, COPD: chronic obstructive pulmonary disease, OPA: obstructive peripheral arteriopathy, BMI: body mass index, CAD: coronary artery disease, E.F: ejection fraction, MR: mitral regurgitation, L.A: left atrium, LAD: left anterior descending artery, LCx: circumflex artery, RCA: right coronary artery. NS: not statistically significant (p > 0.05)

**Table 4:** Impact of intra and postoperative parameters on the occurrence of post-CABG atrial fibrillation

Characteristic	Group A (AF)	Group B (no AF)	Significance (p)
	157 patients	191 patients	
**CPB-time**			
< 60 min	12	23	NS
60-120 min	101	136	NS
> 120 min	44	32	p = 0.011

**M.I.I (ml.m^2^/Kg.min)**			
av.MII _ante _≥ 0.5	40	64	NS
av.MII _ante _0.28 - < 0.49	77	99	NS
av.MII _ante _≤ 0.27	40	28	p = 0.011

**CABG**			
1 graft	11	13	NS
2 grafts	46	56	
3 grafts	96	114	
4 grafts	4	8	

**LIMA use**	145	167	NS

**Positive fluid balance**	114	93	p < 0.001

**Potassium deficit**	118	158	NS

**FiO_2_/PO_2_**			
≤ 40	129	173	NS
> 40	28	18	p = 0.021

**Inotropic support**			
No	100	135	NS
3-15 μg/kg/min	24	38	NS
> 15 μg/kg/min	33	18	p = 0.016

**Perioperative MI**	16	3	p < 0.001

**ICU-recovery**			
≤ 48 hours	109	170	NS
> 48 hours	48	21	p < 0.001

CPB: Cardiopulmonary Bypass, M.I.I: Myocardial Ischemia Index, CABG: Coronary Artery Bypass Grafting, LIMA: left internal thoracic (mammary) artery, FiO_2_: fraction of delivered O_2_, PO_2_: arterial partial pressure of O_2_, MI: Myocardial Infarction, ICU: Intensive Care Unit, NS: not statistically significant (p > 0.05)

## Discussion

AF is the result of the dispersion of atrial refractoriness resulting in multiple reentry wavelets in the atria [8]. In the postsurgical state of the heart several parameters may alter the refractoriness of adjacent atrial areas predisposing to reentry circuits and to the development of atrial fibrillation: inflammation [9], heightened sympathetic and vagal stimulation [10,11], fluid overload and postoperative ventricular stunning resulting in atrial pressure elevation [12,13], chronic distention of the left atrium [14,15], metabolic derangements such as hypoglycemia [16] and altered thyroid function, including both hyper- and hypo-thyroidism [17], alterations of the cardiac structure and electrophysiological profile of the atria due to the surgical atrial trauma itself [5], and ischemic atrial injury [18,19].

**Figure 1:**
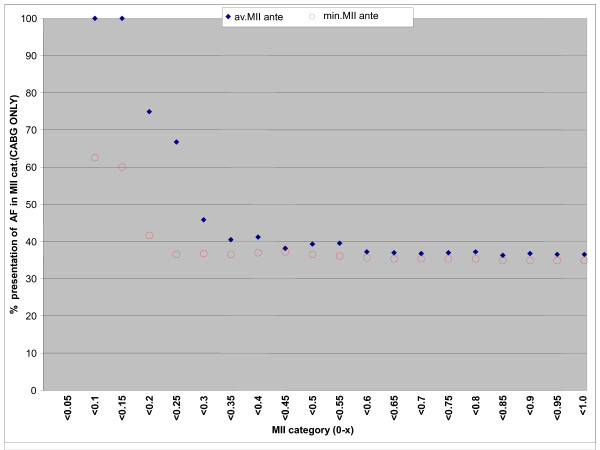
**Distribution of the _av_MII_ante _and _min_MII_ante _values in relation to the frequency of postoperative AF**. Note: _av_MII_ante_: average value of Myocardial Ischemia Index, _min_MII_ante_: minimum value of Myocardial Ischemia Index.

The intraoperative ischemia of the atrial wall has been considered as the most important factor related to the pathophysiological changes resulting in postoperative AF [20]. It has been shown that during a heart operation both the atrial septum and atrial wall remain warmer than the wall of the left ventricle [4,21,22]. Therefore, the protection of the atrial wall remains relatively inadequate compared to that of the left ventricular wall. Based on that consumption several trials have been carried out to identify the impact of different techniques of myocardial protection on the incidence of postoperative atrial arrhythmias without any clear benefit for any of the various strategies applied [23]. In our opinion the amount of cardioplegia is the most important factor related to the postoperative occurrence of AF. Jideus et al [24] showed that larger amounts of cardioplegia are related to lower incidence of postoperative AF. In our cohort of patients we observed a statistically significant relation between myocardial injury and postoperative AF. As shown in Figure 1 describing the distribution of the _av_MII_ante _values in relation to the frequency of postoperative AF, values of _av_MII_ante _< 0.27 mls.m^2^/kg.min were related to a higher incidence of AF after CABG surgery (p = 0.011). Furthermore, when performing the same analysis using the lowest values of the MII_ante _(_min_MII_ante_) we observed that the _av_.MII_ante _was a stronger predictor of postoperative atrial fibrillation than the _min_MII_ante _indicating that one inadequate cardioplegia delivery is less important than more ones (Figure 1).

The prolonged CPB-time in cardiac surgery may result from any one or more of the following factors: delay in first placing the aortic cross clamp, prolonged cardioplegic deliveries, extended warm shot and prolonged reperfusion period, and not just prolonged ischemic intervals. In our study we found that CPB-time above 120 minutes was statistically related to postoperative AF. However, in contrast to other authors [25,26] we haven't found any relation between the aortic cross clamp time and the frequency of postoperative AF. Furthermore, the quality of the coronary arteries and the number of bypasses performed, although reported as factors related to the length of ischemic time, showed no statistical influence on the outcome of postoperative AF.

Intraoperative infarction was statistically related to postoperative AF. This fact is also suggested by other authors [27,28]. In our opinion it is possible that posterior infarcts are directly involved inducing ischemia of the atrial wall and septum while the anterior ones are indirectly implicated through the development of acute atrial enlargement. This last hypothesis is supported by the results of Knotzer et al [29] who observed that post-CABG high filling pressure in both atria due to ventricular stunning are statistically related to an increased incidence of postoperative AF. In the same study it has been shown that systemic hypoxia is also related to the development of postoperative AF. Such observation is also supported by our study. The systemic hypoxia may result from preexisting compromise of the patient's respiratory function with decreased pulmonary reserves or may be related to other parameters such as perioperative myocardial infarction causing interstitial pulmonary edema, or positive fluid balance. Positive fluid balance was found relative to the occurrence of postoperative AF in our study. A plausible explanation is that the positive fluid balance influences the development of AF through higher filling pressures of the left atrium and pulmonary congestion resulting in hypoxia. However, its role as a prognosticator is questionable. Both Osranek et al [15] and Place and colleagues [30] failed to identify net fluid balance either intra-operatively or postoperatively as a significant factor related to AF.

Postoperative low cardiac output has been reported as a parameter statistically related to postoperative AF [31]. In our opinion this observation is the result of the high inotropic support used in these patients to attain sufficient cardiac output. In this study indeed we found that high inotropic support (doses of Dopamine or Dobutamine, > 15 μg/kg/min) was statistically related to the incidence of postoperative AF.

A long ICU stay was found to be statistically related to the occurrence of AF after CABG. However this is a false presumption since a protracted ICU recovery may depend on other factors such us hypoxia, perioperative myocardial infarction and sepsis that predispose the patient to the development of postoperative arrhythmias.

We found that age > 65 years was a significant predictor of AF after CABG. Advanced age has been documented as the most consistent predictor of AF after cardiac surgery [1,2,15,27,28,31-33]. Older patients present alterations in their atrial electrophysiological profile due to degenerative and inflammatory processes and therefore are more susceptible to the development of atrial fibrillation, especially in port cardiac surgery settings [34]. This could also explain why patients with a history of episodes of AF prior to surgery have a greater risk to develop AF after surgery. In this study indeed all patients with episodes of AF within 3 months prior to surgery and AF after CABG belonged to the advanced age group (> 65 years old); on the contrary most patients with early preoperative onset AF and without post-CABG AF were less than 65 years old.

Both low ejection fraction and congestive heart failure prior to surgery have been recognized as independent predictors of AF [2,4,35]. These conditions result in chronic retention of blood in the atria, dilation of the atrial chambers and enlargement of their walls, providing an excellent substrate for the development of reentry circuits in the presence of intraoperative ischemia. This observation was also valid in our study, where an ejection fraction lower than 40% was statistically related to the incidence of AF after surgery.

Furthermore, we observed that patients presenting proximal lesions to the right coronary artery showed an increased incidence of AF which was statistically significant. Similar observations were made by Mendes et al [36] and Kolvekar and colleagues [19], supporting the role of diseased sino-atrial node and atrio-ventricular node arteries originating from the RCA in the development of AF.

Finally, patients suffering from COPD were at higher risk to develop AF. A plausible explanation is that patients suffering from impaired respiratory function are more likely to present hypoxia postoperatively especially if more contributing factors such as positive fluid balance, increased pulmonary artery and atrial pressures, perioperative myocardial infarction, lung atelectasis, infection and lung dysfunction related to the cardiopulmonary bypass, coexist.

## Conclusions

Based on our results the incidence of postoperative atrial fibrillation can be predicted by specific preoperative and perioperative parameters. Advanced age represents an optimal substrate for the development of the arrhythmia especially when combined with increased stress of the atrial wall. This stress may result from chronic stress to the atrial wall such as the one observed in patients with low ejection fraction and congestive heart failure, intraoperative ischemic injury and postoperative stress factors like myocardial infarction and positive fluid balance. Intraoperative ischemic injury is sufficiently expressed by the M.I.I which is related to the magnitude of atrial mass (approximated here by the BMI), the amount of cardioplegia delivered and the time between the cardioplegic deliveries. M.I.I represents an excellent predictor of postoperative AF after conventional coronary artery surgery. Patients presenting such predictors of AF may benefit from the precautionary early commencement of anti-arrhythmic treatment.

## Competing interests

The authors declare that they have no competing interests.

## Authors' contributions

All authors: 1) have made substantial contributions to conception and design, or acquisition of data, or analysis and interpretation of data; 2) have been involved in drafting the manuscript or revising it critically for important intellectual content; and 3) have given final approval of the version to be published.
